# The Prediction of Acute Postoperative Pain Based on Neural Oscillations Measured before the Surgery

**DOI:** 10.1155/2021/5543974

**Published:** 2021-04-09

**Authors:** Qi Han, Lupeng Yue, Fei Gao, Libo Zhang, Li Hu, Yi Feng

**Affiliations:** ^1^Department of Anesthesiology, Peking University People's Hospital, Beijing, China; ^2^CAS Key Laboratory of Mental Health, Institute of Psychology, Chinese Academy of Sciences, Beijing, China; ^3^Department of Psychology, University of Chinese Academy of Sciences, Beijing, China; ^4^Department of Pain Medicine, Peking University People's Hospital, Beijing, China

## Abstract

Even with an improved understanding of pain mechanisms and advances in perioperative pain management, inadequately controlled postoperative pain remains. Predicting acute postoperative pain based on presurgery physiological measures could provide valuable insights into individualized, effective analgesic strategies, thus helping improve the analgesic efficacy. Considering the strong correlation between pain perception and neural oscillations, we hypothesize that acute postoperative pain could be predicted by neural oscillations measured shortly before the surgery. Here, we explored the relationship between neural oscillations 2 hours before the thoracoscopic surgery and the subjective intensity of acute postoperative pain. The spectral power density of resting-state beta and gamma band oscillations at the frontocentral region was significantly different between patients with different levels of acute postoperative pain (i.e., low pain vs. moderate/high pain). A positive correlation was also observed between the spectral power density of resting-state beta and gamma band oscillations and subjective reports of postoperative pain. Then, we predicted the level of acute postoperative pain based on features of neural oscillations using machine learning techniques, which achieved a prediction accuracy of 92.54% and a correlation coefficient between the real pain intensities and the predicted pain intensities of 0.84. Altogether, the prediction of acute postoperative pain based on neural oscillations measured before the surgery is feasible and could meet the clinical needs in the future for better control of postoperative pain and other unwanted negative effects. The study was registered on the Clinical Trial Registry (https://clinicaltrials.gov/ct2/show/NCT03761576?term=NCT03761576&draw=2&rank=1) with the registration number NCT03761576.

## 1. Introduction

More than 230 million major surgeries are performed annually around the world [1]. Even with an improved understanding of pain mechanisms and advances in perioperative pain management, inadequately controlled postoperative pain continues. In fact, the incidence of moderate-to-severe acute postoperative pain ranges from 20% to 80% [2]. Postoperative pain would lead to a series of negative outcomes, including delayed recovery time, increased cost of care, and an increased incidence of the transition from acute pain to chronic pain [1]. One way to prevent these issues is to adopt more effective analgesic strategies in the perioperative period. More effective analgesia could be achieved with the help of successful predictions of acute postoperative pain based on physiological measures before the surgery. Exploiting the power of machine learning techniques, we could identify patients at risk by predicting acute postoperative pain based on physiological measures before the surgery. The correct prediction would help deepen our understanding of the biological underpinning of the risk. In addition, the prediction would help develop more targeted or preventative treatments (i.e., individualized treatments) to improve analgesic efficacy [3, 4].

Pain is a sensory and emotional experience with a high level of variability across different individuals [5]. Importantly, pain perception is closely related to neural oscillations [6], which play a crucial role in the segregation and integration of brain regions for functioning [7]. Specifically, numerous studies of acute and chronic pain using electroencephalography (EEG) and magnetoencephalography (MEG) highlighted the important role of neural oscillations at theta, alpha, beta, and gamma frequency bands in characterizing pain perception [6, 8–13], although the specificity of the relationship between neural oscillations and pain is disputed. The relationship between pain perception and neural oscillations could be summarized in three main aspects. First, nociceptive stimuli can induce significant modulations of neural oscillations at theta, alpha, beta, and gamma frequency bands [5, 6, 13, 14], and importantly, the changes of the magnitude of some neural oscillations are robustly correlated with the subjective intensity of pain perception [5, 13, 15]. For example, gamma oscillations recorded from the primary somatosensory cortex could predict the subjective pain intensity within the same individual and encode pain sensitivity across different individuals [5]. In addition, gamma oscillations in the prefrontal cortex are likely to encode subjective pain perception of tonic heat pain [10, 16]. Second, neural oscillations before nociceptive stimuli could also predict the subjective intensity of pain perception that is evoked by the forthcoming nociceptive stimuli [17, 18]. Specifically, alpha oscillations at bilateral central regions and gamma oscillations at parietal regions act synergistically and causally in predicting the intensity of pain perception elicited by subsequent nociceptive stimuli [17]. Third, altered neural oscillations are observed in many chronic pain conditions, such as fibromyalgia [8, 13], chronic back pain [9], and postherpetic neuralgia [12]. For example, one MEG study found that fibromyalgia patients exhibited increased beta and gamma power in the dorsolateral prefrontal and orbitofrontal cortex [8]. Interestingly, increased power in these two frequency bands also correlated with higher affective pain scores in fibromyalgia patients [8].

Considering the strong correlation between pain perception and neural oscillations, we hypothesize that acute postoperative pain could be predicted by neural oscillations measured shortly before the surgery. In practice, this research aim could be achieved based on the combination of neuroimaging techniques to record neural oscillations and machine learning techniques [19, 20] to predict postoperative pain. Machine learning is referred to as a set of algorithms that can automatically detect patterns from neuroimaging data and utilize the detected patterns to predict clinical outcomes [21, 22], e.g., the intensity of acute postoperative pain. Accumulating evidence has been documented to show that machine learning is able to extract meaningful information from high-dimensional and noisy neuroimaging data, thus effectively identifying neural markers for behaviors and diseases [21, 22].

Here, neural oscillations were measured using the EEG technique shortly before the surgery, for which the technique is clinically feasible in most situations as EEG can be used directly at the patients' bedside. The relationship between neural oscillations before the surgery and the subjective intensity of acute postoperative pain was quantified using spectral analysis and partial correlation analysis. Afterward, machine learning techniques were applied to predict the intensity of postoperative pain based on EEG recordings shortly before the surgery. Ideally, surgery patients with a high risk of postoperative pain could be identified before the surgery, which could provide a vital measure to optimize the analgesic strategy for better control of the postoperative pain and other negative outcomes.

## 2. Methods

The clinical study was conducted at the Department of Anesthesiology of the Peking University People's Hospital, Beijing, China. The Medical Ethics Committee of the Peking University People's Hospital approved the study protocol. A written informed consent was obtained from all participants. The study was registered on the Clinical Trial Registry (https://register.clinicaltrials.gov/) with the registration number NCT03761576.

### 2.1. Participants

Patients admitted to the Peking University People's Hospital for lobectomy, wedge resection, or mediastinotomy under thoracoscopic surgery were recruited between November 2018 and March 2019. Inclusion criteria were (1) age between 35 and 65 years, (2) education levels beyond secondary school, (3) American Society of Anesthesiologist (ASA) grade I-II, (4) preferred to use postoperative patient-controlled analgesia (PCA), and (5) signed informed consent. Exclusion criteria were (1) neurological diseases, (2) psychiatric diseases or psychiatric family history, (3) traumatic brain injury or postcraniotomy, (4) chronic pain suffers or preoperative opioid users, and (5) thoracotomy needed or planned to return to Intensive Care Unit after surgery. The detailed demographic information is summarized in Table 1.

### 2.2. Study Design

**Figure 1:**
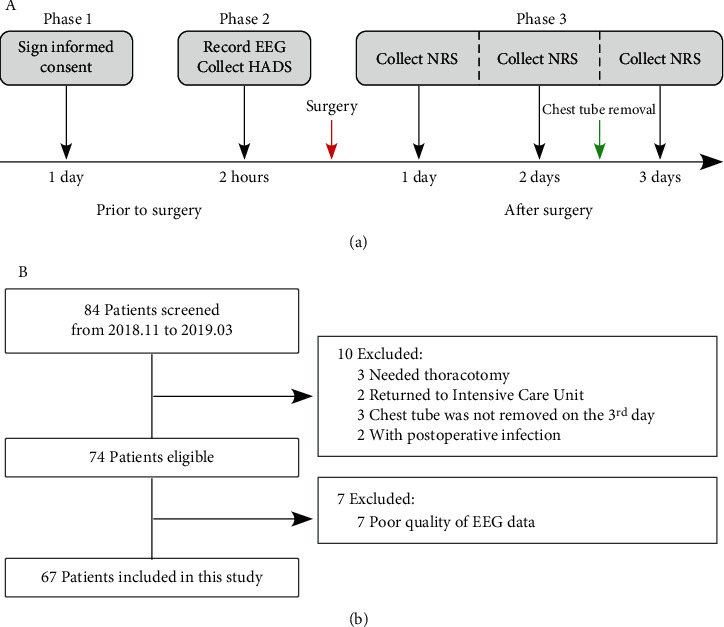
(a) Study design and the (b) flow of participants.

As showed in Figure 1(a), the study design is composed of three phases. In phase 1, patients were instructed to sign the informed consent one day before their surgery, and patients were required to avoid smoking or drinking coffee or caffeine-containing beverages 10 hours before the surgery. In phase 2, resting-state EEG data were collected from all patients 2 hours before the surgery (please see the following section for details about EEG data collection), and all patients were instructed to complete the Hospital Anxiety and Depression Scale (HADS) before EEG data collection. In phase 3, postoperative pain on the 1^st^, 2^nd^, and 3^rd^ days after the surgery was collected from all patients. Specifically, the highest postoperative pain over the past 24 hours was assessed on an 11-point numerical rating scale (NRS) (0 = no pain, 10 = worst pain imaginable) at 10-14 o'clock on the 1^st^ and 2^nd^ days after the surgery. On the 3^rd^ day after the surgery, the highest postoperative pain over the past 24 hours was assessed using the same NRS, but after the chest analgesic tube was removed. Please note that the highest postoperative pain over the past 24 hours was obtained by the evaluation of pain at rest and pain due to movements, e.g., coughing and breathing. As recommended by Zalon in 2014 [23], clinicians should actively intervene with patients with a pain score (i.e., NRS scores) more than 3. For this reason, patients with NRS scores higher than 3 on the 3^rd^ day after the surgery were considered with moderate/high pain, while patients with NRS scores of 3 or lower than 3 were considered without low pain. All patients were examined by the same investigator, and all patients were reminded that they could withdraw from the experiment at any time for any reason, but none did so.

Please note that after EEG data collection, patients first received local anesthesia (i.e., thoracic paravertebral block at T4 and T7 on the affected side) and then received general anesthesia according to the local clinical standards to ensure the safety of the surgery. Patients received thoracic paravertebral block at T4 and T7 on the affected side with an infusion of 0.4% bupivacaine (20 ml) before general anesthesia. The induction of anesthesia was achieved using midazolam (0.02-0.04 mg/kg), propofol (1-2 mg/kg), and sufentanil (0.2-0.4 *μ*g/kg). Then, rocuronium (0.6-1 mg/kg) was intubated with a double-lumen endotracheal tube, for which the position was confirmed by fiberoptic bronchoscopy. Anesthesia was maintained with 1% sevoflurane, propofol (0.1-0.3 *μ*g/kg/min), and remifentanil (0.1–0.3 *μ*g/kg/min) during the surgery. More sufentanil and rocuronium were supplied when needed, and the total amount of sufentanil should be no more than 0.6 *μ*g/kg. Flurbiprofen axetil (100 mg) was started to be administrated 30 min before the end of the surgery. Along with the infusion of flurbiprofen axetil (8 mg/h), the patient-controlled analgesia (PCA) with oxycodone (Perfusor fm PCA; single dose 1 mg, lockout 5 min, limit 8 mg/h) was used for all patients as soon as they were able to operate the system.

#### 2.2.1. EEG Recording

Patients lay in a bed with a semirecumbent position in a silent, temperature-controlled room. The EEG cap was mounted on their head with conducting gel inserted for each electrode, and all electrode impedances were kept lower than 10 k*Ω*. EEG data were recorded using a 32-channel NuAmps Quickcap, NuAmps DC amplifier, and Scan 4.5 Acquisition software (Compumedics Neuroscan, Inc. Charlotte, NC, USA). The NuAmps amplifier (Model 7181) was set with a sampling rate of 1000 Hz and with a signal bandpass filter from 0.01 to 100 Hz. The ground electrode was positioned 10 mm anterior to Fz, and the right mastoid electrode (M2) was used as the online reference. During EEG data collection (five minutes in total), all subjects were instructed to keep awake, relaxed, and eyes closed, since the test-retest reliability of resting-state EEG data was higher in the eyes closed condition than in the eyes open condition [24].

#### 2.2.2. EEG Preprocessing

EEG data were preprocessed using EEGLAB [25]. Continuous EEG data were first offline rereferenced to the average bilateral mastoid electrodes (M1 and M2). Then, EEG data were bandpass filtered between 0.5 and 80 Hz and notch filtered between 48 and 52 Hz. For the artifact rejection, continuous EEG data were segmented into epochs using a time window of 5 s. EEG epochs were decomposed into a series of independent components (ICs) using the infomax algorithm as implemented in EEGLAB [25]. The number of ICs was equal to the number of EEG electrodes. ICs contaminated by eye blinks and movements were identified and removed using the SASICA algorithm [26, 27]. The number of the removed ICs was comparable for the low-pain and moderate/high-pain groups (2.7 ± 0.22 and 2.3 ± 0.21, respectively, *p* = 0.19). Moreover, epochs contaminated by gross artifacts (i.e., exceeding ±75 *μ*V in any channel) were automatically rejected. The proportion of epochs rejected was not significantly different between the low-pain and moderate/high-pain groups (19 ± 4.5% and 22 ± 3.9%, respectively, *p* = 0.6).

#### 2.2.3. EEG Spectral Analysis

For each patient, the preprocessed EEG data were transformed to the frequency domain using Welch's method (window length: 2 s; overlap: 50%) [28], yielding an EEG spectrum ranging from 0.5 to 80 Hz, in steps of 0.5 Hz. Group-level EEG spectra were obtained by calculating the average of single-patient EEG spectra in each group (i.e., moderate/high-pain group and low-pain group). To assess the group difference of EEG spectra, a point-by-point independent-sample *t*-test was performed for each frequency (across all frequency bins) and each electrode, and the significant level (*p* value) was corrected using a false discovery rate (FDR) procedure [29]. Additionally, to control for false-positive observations, the frequency intervals with a *p* value smaller than the defined threshold (*p*_fdr_ < 0.05) for more than 5 Hz were considered as significant. Partial correlation analysis was also performed between EEG power at different frequency bands and acute postoperative pain (i.e., NRS scores on the 3^rd^ day after the surgery) to assess their relationship while controlling for the effect of age and removing the possible outliers. Please note that the outliers were identified using the threshold of three standard deviations of EEG power, i.e., the data was identified as an outlier if its value was three standard deviations away from the mean [30].

#### 2.2.4. Machine Learning: Classification and Regression

We performed the linear discriminant analysis (LDA) [31], a typical machine learning algorithm, to predict the intensity of postoperative pain based on EEG recordings shortly before the surgery. Considering the arbitrary nature of dichotomizing the two groups, we also predicted the continuous pain ratings (i.e., the intensity of postoperative pain) using the multiple linear regression (MLR) [32]. Leave-One-Out Cross-Validation (LOOCV) [33] was used to assess the prediction performance. Specifically, LOOCV was achieved by dividing all subjects (*N* subjects) into *N* − 1 training subjects and 1 test subject, and the same procedure was repeatedly performed *N* times to ensure that every subject was used as the test subject once. The classification accuracy and correlation coefficient (*R*) between the real pain intensities and the predicted pain intensities were used to evaluate the prediction performance of LDA and MLR, respectively.

To assess the contribution of EEG feature at each electrode and each frequency on the prediction performance, the LDA and MLR were firstly performed for each electrode in the spatial domain and each frequency in the frequency domain. For both classification and regression, all EEG features were tested once, and the maximal values of prediction accuracy for classification and correlation coefficient for regression at the electrode level and the frequency level were, respectively, used to evaluate the contribution of these features in the machine learning model.

To achieve better prediction performance, EEG features at all electrodes and all frequencies (i.e., the combination of features at the spatial and frequency domains) were used in the multivariate machine learning model. In the present study, there were 160 features for each electrode (from 0.5 Hz to 80 Hz with a resolution of 0.5 Hz) and 30 electrodes. In total, the feature dimension was 4800 (160 frequency bins × 30 electrodes), and the sample size was 67 (67 patients). This is a typical small sample size pattern recognition problem with a high feature dimension. In this case, the curse of feature dimensionality is the main problem for both classification and regression. To address this issue, feature selection is required before performing prediction. In addition, feature selection is an effective strategy for dimension reduction to prevent overfitting. Here, we firstly shrunk the features in the frequency domain to the range of 20-70 Hz, since there was no significant difference between the two groups for all channels outside the frequency range (i.e., 0.5-20 Hz and 70-80 Hz). Secondly, Sequential Floating Forward Selection (SFFS) method was used as a wrapper approach for additional feature selection [29]. As a heuristic search method [34], the SFFS algorithm starts with an empty feature set and mainly consists of a forward step for inserting features and a backward step for deleting features. The forward step searches the best features outside the feature set to improve the prediction performance in the cross-validation. After each forward step, the backward step removes the feature in the feature set as long as the performance could be improved in the cross-validation. The whole process of the SFFS would stop if the prediction performance could not be improved or the feature dimension reaches 50.

#### 2.2.5. Statistical Analysis

Demographic information of patients in the moderate/high-pain group and the low-pain group was compared using chi-square tests (i.e., gender, education level, ASA grade, and operation type) and an independent-sample *t*-test (i.e., age). Group differences in HADS scores (i.e., anxiety score and depression score), doses of oxycodone, and postoperative pain (i.e., NRS scores on the 1^st^, 2^nd^, and 3^rd^ days after the surgery) were evaluated using independent-sample *t*-tests. All statistical analyses were carried out in SPSS 25.0 (SPSS Inc., New York, USA), and the statistical significance level was set at 0.05.

## 3. Results

We screened 84 patients undergoing thoracoscopic surgery for eligibility. As summarized in Figure 1(b), 10 patients were excluded from the study for the following reasons: 3 patients needed thoracotomy, 2 patients returned to the Intensive Care Unit after the surgery, 3 patients for whom the chest analgesic tube was not removed on the 3^rd^ day after the surgery, and 2 patients with postoperative infection (i.e., the body temperature was higher than 38.0°C for more than 2 days). Moreover, data from 7 patients were excluded from the following analysis due to the poor quality of EEG data. As a result, 67 patients were eligible for inclusion. According to the postoperative pain on the 3^rd^ day after the surgery, the eligible patients were assigned to the two groups: moderate/high-pain group (*n* = 33) and low-pain group (*n* = 34). As demonstrated in Table 1, no significant differences were observed between patients in the moderate/high-pain group and those in the low-pain group for clinical and demographic characteristics, i.e., gender, age, education level, ASA grade, and operation type. In addition, both anxiety and depression scores, as evaluated using the HADS, were not significantly different between patients in the two groups. However, the dose of oxycodone and postoperative pain (e.g., NRS on the 2^nd^ day after the surgery) were significantly higher for patients in the moderate/high-pain group than those in the low-pain group (*p* = 0.021 and *p* < 0.001, respectively).

**Figure 2:**
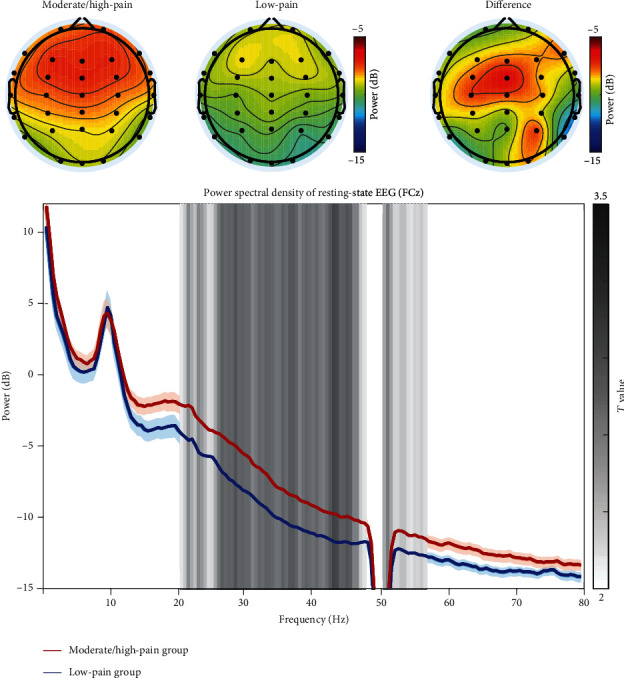
The difference of spectral power density of resting-state neural oscillations between patients with different levels of acute postoperative pain, i.e., moderate/high pain and low pain.

**Figure 3:**
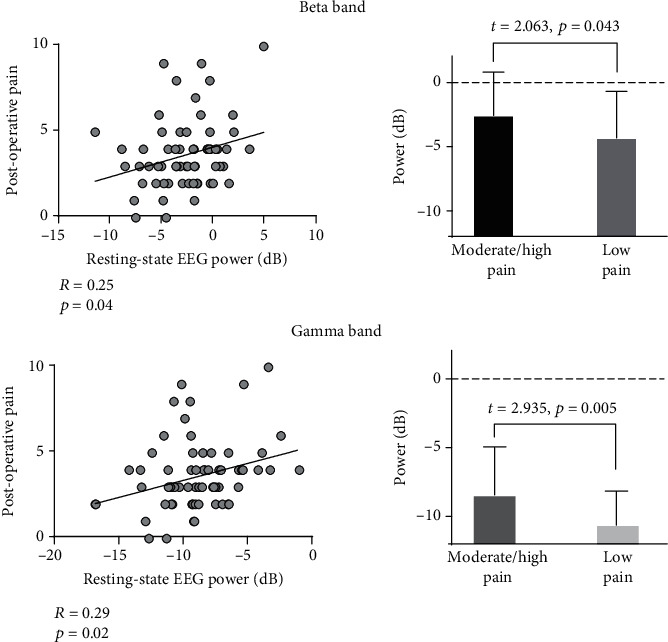
The relationship between postoperative pain and resting-state EEG powers at beta (14-30 Hz) and gamma (31-50 Hz) frequency bands. *Left*: partial correlation was performed after controlling age and removing outliers. *Right*: independent-sample *t*-tests were performed between patients with different levels of acute postoperative pain, i.e., moderate/high pain and low pain.

Group-level spectra of resting-state EEG oscillations in both moderate/high-pain and low-pain groups are showed in Figure 2. The point-by-point statistical analysis revealed that patients in the moderate/high-pain group had significantly higher spectral power density of resting-state EEG oscillations at the frontocentral region (maximal at FCz electrode) within beta and gamma frequency bands (between 21 and 55 Hz) than patients in the low-pain group (*p*_fdr_ < 0.05). When taken separately, beta (14-30 Hz) and gamma (31-50 Hz) band powers in the moderate/high-pain group were both significantly higher than those in the low-pain group (see Figure 3; beta band: *t* = 2.063, *p* = 0.043; gamma band: *t* = 2.935, *p* = 0.005). To test the robustness of results, we also performed partial correlation analysis between EEG power at beta and gamma frequency bands and acute postoperative pain (i.e., NRS scores on the 3^rd^ day after the surgery) while controlling for the effect of age and removing the possible outliers which lay three standard deviations away from the mean. Both beta band power (partial *R* = 0.25, *p* = 0.04) and gamma band power (partial *R* = 0.29, *p* = 0.02) were significantly correlated with acute postoperative pain (Figure 3).

**Figure 4:**
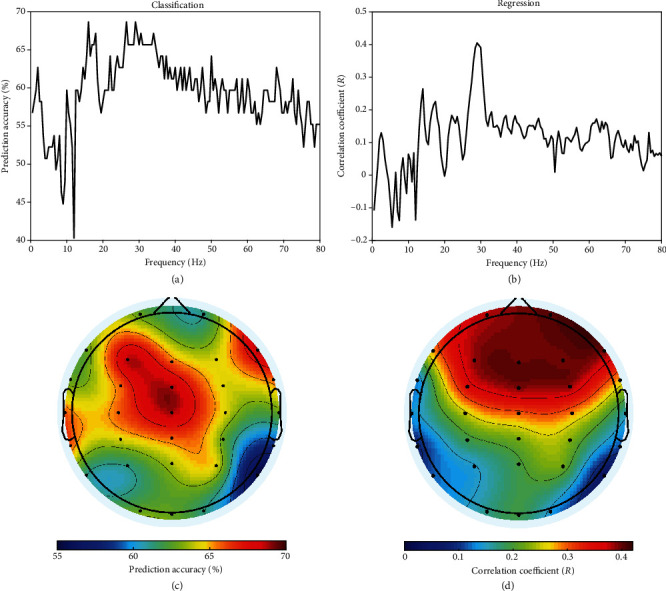
The contribution of presurgery EEG features on the performance of classification and regression to predict the intensity of postoperative pain ((a, b) in the frequency domain; (c, d) in the spatial domain).

The contribution of presurgery EEG features on the performance of machine learning algorithms to predict the intensity of postoperative pain is displayed in Figure 4. EEG features in the frequency domain showed distinct patterns of contribution to the prediction performance for classification (i.e., LDA, Figure 4(a)) and regression (i.e., MLR, Figure 4(b)). However, the features around 30 Hz provided the most discriminative information for both classification and regression. As displayed in Figures 4(c) and 4(d), EEG features in the frontocentral region were more discriminative for both classification and regression. For classification, electrodes Cz, FCz, and F3 provided the highest prediction accuracy. For regression, electrodes F3, F4, FCz, and Fz provided the highest correlation coefficient.

**Figure 5:**
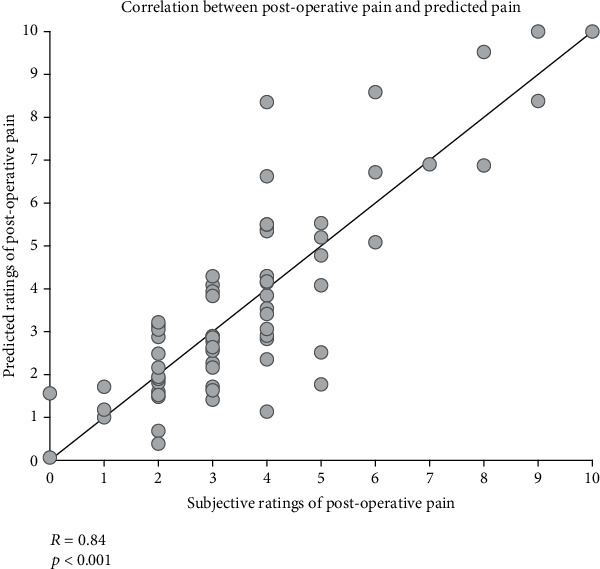
The correlation between subjective ratings of postoperative pain and the predicted ratings of postoperative pain by multiple linear regression with LOOCV.

To achieve better prediction performance, EEG features at all electrodes and all frequencies (i.e., the combination of features at the spatial and frequency domains) were used in the multivariate machine learning model. The SFFS algorithm was stopped with 50 extracted features for both classification and regression. For classification, LDA with 50 features provided a prediction accuracy of 92.54% in the LOOCV test, i.e., 5 out of 67 patients have been misclassified. For regression, the MLR with 50 features also showed good prediction performance (Figure 5), i.e., the correlation between the real pain intensities and the predicted pain intensities was very strong (*R* = 0.84, *p* < 0.001). These results suggested that the combination of EEG features at the spatial and frequency domains would provide complementary information to achieve better prediction performance than any single EEG feature.

## 4. Discussion

In the present study, we found that the incidence of moderate-to-high acute postoperative pain after thoracoscopic surgery was high (49%) according to the subjective reports of pain intensity on the 3^rd^ day after the surgery. EEG data collected 2 hours before the surgery showed that patients in the moderate/high-pain group had significantly higher spectral power density of resting-state beta and gamma band oscillations at the frontocentral region than patients in the low-pain group (Figure 2). In addition, a positive correlation was observed between the spectral power density of resting-state beta and gamma band oscillations and subjective reports of postoperative pain (Figure 3). Importantly, applying machine learning technique, the intensity of acute postoperative pain could be accurately predicted based on the recorded resting-state EEG data before the surgery (prediction accuracy is 92.54%, and the correlation coefficient between the real pain intensities and the predicted pain intensities is 0.84). Therefore, our study provided a feasible strategy to identify patients with a high risk of postoperative pain before the surgery using EEG recordings. This strategy could hopefully meet the clinical needs in the future as it would help optimize the analgesic strategy for better control of postoperative pain and other unwanted negative effects.

With the improved understanding of pain analgesic mechanisms, more and more analgesia techniques have been developed to manage postoperative pain [35–38]. Although multimodal analgesia is normally applied in clinical practice, clinical pain assessment based on subjective pain reports, behavioral assessment tools, or solicit input from caregivers [39] suggests that control of postoperative pain is still inadequate [38, 40]. One survey showed that approximately 75% of patients still reported pain after discharge [41]. In the present study, approximately 49% of surgery patients experienced moderate-to-high acute postoperative pain (on the 3^rd^ day after the surgery), for which the incidence of postoperative pain was similarly high in previous studies [40–42]. Please note that significant difference of postoperative pain was already observed on the 2^nd^ day after the surgery between the moderate/high-pain group and the low-pain group. The inadequate management of postoperative pain calls for more effective analgesic strategies. Successful prediction of acute postoperative pain using physiological measures before the surgery would offer valuable information on who needs to be treated preemptively, thus paving the way for more effective and individualized analgesia.

In the present study, the analysis of resting-state EEG data collected 2 hours before the surgery showed that the spectral power density of beta and gamma band oscillations at the frontocentral region was able to distinguish patients with different levels of acute postoperative pain. Additionally, the spectral power density of resting-state beta and gamma band oscillations was positively correlated with the subjective report of postoperative pain.

A series of neural oscillations plays an important role in encoding pain perception for both healthy and disease conditions [8, 15, 16, 43–45]. Evidence showed that beta band oscillations are highly associated with sensorimotor functions, e.g., the preparation before the movement and the calibration during the movement [46, 47]. For instance, beta band oscillations enhance when the movement is inhibited or voluntarily suppressed [46]. There is a close relationship between movement and pain, and nocifensive behaviors that responded to pain have important protective functions for humans [48]. In addition to movement-related functions, beta band oscillations have been shown to be highly associated with pain in both basic and clinical conditions. In healthy subjects, the power of beta band oscillations has been shown to be modulated by acute pain that was elicited by electrical, laser, and contact heat stimuli [15, 49, 50]. However, it is important to note that the functional interpretation of beta band oscillations is still speculative since, in this study, EEG data were collected before the surgery, and no pain-related movement could be observed at this time. Future studies are required to delineate the detailed mechanism behind the association between the spectral power density of beta band oscillations and acute postoperative pain.

Gamma band oscillations, which are believed to play a crucial role in cortical integration and perception [6, 49, 51], reflect cortical activity directly related to pain perception [16, 51, 52]. For this reason, gamma band oscillations are currently one of the most promising biomarkers of pain perception [5, 51]. Importantly, the solid relationship between gamma band oscillation and pain perception was observed not only at the within-subject level but also across different subjects, in both humans [5, 51] and rodents [14]. In terms of mechanism, the close relationship between gamma band oscillation and pain perception would be associated with the integrating role of gamma band oscillations in the generation of the conscious experience of pain [53], as gamma band oscillations are important for communications within a large network of cortical and subcortical brain regions [17, 54]. Moreover, gamma band oscillations have been demonstrated to subserve a filtering mechanism to select behaviorally relevant information for action [16, 52]. In the present study, we observed that the spectral power density of gamma band oscillations was positively correlated with the subjective report of postoperative pain. This observation provided additional evidence for the close relationship between gamma band oscillation and pain perception across different human subjects and might be related to the possible communication between distributed neuronal ensembles for subsequent pain-related behaviors [5, 11].

Also, our results showed that pain-related beta and gamma band oscillations were mostly recorded from the frontocentral region. This is reminiscent of previous studies on acute and chronic pain [8, 9, 12, 16]. Some researchers posited that pain chronification involves a shift from brain circuits associated with sensory processes to emotional circuits [55]. Supporting this idea, one previous study showed that beta and gamma band power increases in the dorsolateral prefrontal and orbitofrontal cortex were correlated with higher affective pain scores in fibromyalgia patients [8]. In addition, the prefrontal cortex is generally believed to be involved in emotion processing [56]. The tonic and ongoing nature of clinical pain resembles that of chronic pain better than phasic pain, like transient laser-induced thermal pain, in which the somatosensory cortex seems to play crucial roles [5]. The importance of prefrontal oscillations, therefore, may suggest that the emotional processing in patients who tend to develop postoperative pain is already somewhat compromised in the presurgery stage, even though these patients may display no explicit emotional disorders or manifestly abnormal emotional processing. Future studies are needed to test whether emotion does play some roles in the relationship between neural oscillations and the probability of developing postoperative pain. It should be noted that no significant correlation between alpha oscillations and acute postoperative pain was observed in the present study. Since alpha oscillations are highly sensitive to the state of the patients (e.g., attention and anticipation), it might be possible that alpha oscillations could be able to predict the intensity of pain perception within a short period of time (e.g., several seconds) [17], but not within a long period of time (e.g., several days in the present study).

The relationship between neural oscillations and acute postoperative pain provided a solid basis for the prediction of postoperative pain with physiological measures before the surgery. The use of machine learning techniques (i.e., LDA with SFFS) achieved a prediction accuracy of 92.54% and a correlation coefficient of 0.84 based on resting-state EEG activities that were recorded 2 hours before the surgery. Ideally, the combination of resting-state EEG recording technique and machine learning algorithms would yield diagnostic biomarkers of acute postoperative pain. This diagnostic biomarker would be important for the development of effective analgesic strategies in the perioperative period, which would be helpful in controlling postoperative pain in surgery patients [21, 22, 53]. In practice, the EEG device is portable and widely equipped, which enables the feasibility in most clinical situations for the application of an EEG-based diagnostic biomarker. Nevertheless, this study did not detangle the respective contribution of trait and state characteristics of patients to the prediction success since EEG data collected 2 hours before the surgery may reflect both characteristics. Importantly, both of them could contribute to the individual difference in analgesic efficacy. If it were trait characteristics that mattered, specifically targeting patients with those traits may be more beneficial and cost-effective. If it were state characteristics, interventions manipulating those states might help achieve better analgesic efficacy.

Several additional limitations in the present study should be noted. First, the current clinical experimental design would possibly be confounded by some unwanted factors. Although a standardized operative procedure was adopted for all patients, there were some variations during the surgery (e.g., the duration and complexity of the surgery and the types and amounts of analgesics), which might also affect the acute postoperative pain. Second, the sample size of the present study is limited, and the age, the educational level, and the type of surgery varied a lot among subjects. In addition, more patients scheduled for other types of surgery should be considered in future studies to verify the identified EEG-based biomarker of postoperative pain. Third, this study did not testify the specificity of EEG oscillations to acute pain. Some studies suggest that gamma oscillations selectively encode phasic and tonic thermal pain [5, 16], but this issue is still in a heated dispute. More importantly, this study did not explicitly address this issue, even though neural oscillations were demonstrated to predict postoperative pain. Future research may test the specificity of EEG oscillations and offer further insights into specifically predicting postoperative pain with neural oscillations or other physiological measures.

In conclusion, we provided solid evidence for the close relationship between the spectral power density of resting-state beta and gamma band oscillations at the frontocentral region and subjective reports of postoperative pain. Moreover, we predicted the level of acute postoperative pain based on features of neural oscillations using machine learning techniques, which achieved a prediction accuracy of 92.54% and a correlation coefficient between the real pain intensities and the predicted pain intensities of 0.84. Therefore, the prediction of acute postoperative pain based on neural oscillations measured before the surgery is feasible and could hopefully meet the clinical needs in the future, thus helping optimize the analgesic strategy for better control of postoperative pain and other unwanted negative effects.

## Figures and Tables

**Table 1:** Participant demographic information and postoperative pain.

Variables	Categories	Moderate/high-pain group (*n* = 33)	Low-pain group (*n* = 34)	*p* value
Gender	Male	14	15	0.729
	Female	20	18	
Age (year)		55.09 ± 6.14	56.45 ± 7.67	0.086
Education level	Junior	6	7	0.277
	High school	13	5	
	College	15	21	
ASA grade	I	13	17	0.281
	II	21	16	
Operation type	Thoracoscopic wedge resection	15	15	0.658
	Thoracoscopic lobectomy	15	16	
	Thoracoscopic mediastinotomy	4	2	
HADS score	Anxiety score	5.06 ± 3.06	4.3 ± 3.02	0.458
	Depression score	5.61 ± 3.79	5.65 ± 3.65	0.506
Dose of oxycodone (mg)		14.03 ± 12.03	8.09 ± 7.44	0.021^∗^
NRS on the 1^st^ day		5.21 ± 1.76	4.41 ± 1.81	0.072
NRS on the 2^nd^ day		5.33 ± 1.93	3.53 ± 1.99	<0.001^∗∗^
NRS on the 3^rd^ day		5.18 ± 1.76	2.24 ± 0.86	/

^∗^ *p* < 0.05; ^∗∗^*p* < 0.001.

## Data Availability

Email to doctor_yifeng@sina.com.
